# Supplementary material for: HIVprotI: an integrated web based platform for prediction and design of HIV proteins inhibitors
Source: J Cheminform. 2018 Mar 9;10:12. doi: 10.1186/s13321-018-0266-y (PMC5845081; doi:10.1186/s13321-018-0266-y)
Supplement: Supplementary file 2 — Additional file 2. Source code of HIVProtI web server. [file 13321_2018_266_MOESM2_ESM.zip › HIVprotI_Source-code/hivprotI/weka-3-6-6/doc/weka/clusterers/package-summary.html]

weka.clusterers


---


|  |  |  |
| --- | --- | --- |
| **Overview** \| **Package** \| Class \| **Tree** \| **Deprecated** \| **Index** \| **Help** \| **Weka's home** | |  |
| **PREV PACKAGE**   **NEXT PACKAGE** | **FRAMES**    **NO FRAMES**     **All Classes** |  |


---

## Package weka.clusterers

| **Interface Summary** | |
| --- | --- |
| **Clusterer** | Interface for clusterers. |
| **DensityBasedClusterer** | Interface for clusterers that can estimate the density for a given instance. |
| **NumberOfClustersRequestable** | Interface to a clusterer that can generate a requested number of clusters |
| **UpdateableClusterer** | Interface to incremental cluster models that can learn using one instance at a time. |

| **Class Summary** | |
| --- | --- |
| **AbstractClusterer** | Abstract clusterer. |
| **AbstractDensityBasedClusterer** | Abstract clustering model that produces (for each test instance) an estimate of the membership in each cluster (ie. |
| **CheckClusterer** | Class for examining the capabilities and finding problems with clusterers. |
| **CLOPE** | Yiling Yang, Xudong Guan, Jinyuan You: CLOPE: a fast and effective clustering algorithm for transactional data. |
| **ClusterEvaluation** | Class for evaluating clustering models. Valid options are: -t name of the training file   Specify the training file. |
| **Cobweb** | Class implementing the Cobweb and Classit clustering algorithms.    Note: the application of node operators (merging, splitting etc.) in terms of ordering and priority differs (and is somewhat ambiguous) between the original Cobweb and Classit papers. |
| **DBScan** | Martin Ester, Hans-Peter Kriegel, Joerg Sander, Xiaowei Xu: A Density-Based Algorithm for Discovering Clusters in Large Spatial Databases with Noise. |
| **EM** | Simple EM (expectation maximisation) class.    EM assigns a probability distribution to each instance which indicates the probability of it belonging to each of the clusters. |
| **FarthestFirst** | Cluster data using the FarthestFirst algorithm.    For more information see:    Hochbaum, Shmoys (1985). |
| **FilteredClusterer** | Class for running an arbitrary clusterer on data that has been passed through an arbitrary filter. |
| **HierarchicalClusterer** | Hierarchical clustering class. |
| **MakeDensityBasedClusterer** | Class for wrapping a Clusterer to make it return a distribution and density. |
| **OPTICS** | Mihael Ankerst, Markus M. |
| **RandomizableClusterer** | Abstract utility class for handling settings common to randomizable clusterers. |
| **RandomizableDensityBasedClusterer** | Abstract utility class for handling settings common to randomizable clusterers. |
| **RandomizableSingleClustererEnhancer** | Abstract utility class for handling settings common to randomizable clusterers. |
| **sIB** | Cluster data using the sequential information bottleneck algorithm.    Note: only hard clustering scheme is supported. |
| **SimpleKMeans** | Cluster data using the k means algorithm   Valid options are: |
| **SingleClustererEnhancer** | Meta-clusterer for enhancing a base clusterer. |
| **XMeans** | Cluster data using the X-means algorithm.    X-Means is K-Means extended by an Improve-Structure part In this part of the algorithm the centers are attempted to be split in its region. |

---


|  |  |  |
| --- | --- | --- |
| **Overview** \| **Package** \| Class \| **Tree** \| **Deprecated** \| **Index** \| **Help** \| **Weka's home** | |  |
| **PREV PACKAGE**   **NEXT PACKAGE** | **FRAMES**    **NO FRAMES**     **All Classes** |  |


---
